# Supplementary material for: Are carbon emissions trading and green financial instruments synergistic? -Comprehensive quantitative research based on content analysis
Source: PLoS One. 2024 Mar 7;19(3):e0298601. doi: 10.1371/journal.pone.0298601 (PMC10919601; doi:10.1371/journal.pone.0298601)
Supplement: S1 Data — (ZIP) [file pone.0298601.s001.zip › Data/地级市Z_F工作报告.zip!/╣≤╓▌/░▓╦│╩╨/└┤╫╘╣1⁄2╓┌║┼í╛┬φ┐╦╩2╛▌═°í┐_2016.docx]

各位代表：

现在，我代表市人民政府向大会作工作报告，请予审议，并请市政协委员和其他列席会议的同志提出意见。

一、“十二五”时期工作回顾

“十二五”是我市经济社会发展取得辉煌成就的时期，是综合实力提升最快、城乡面貌变化最大、生态建设成效最好、改革开放活力最强、人民群众实惠最多的时期。在省委、省政府和市委的坚强领导下，在市人大、市政协的监督支持下，我们始终坚守发展和生态两条底线，坚持主基调主战略，紧紧围绕提速发展、后发赶超、同步小康总目标，全力推进工业化、城镇化、农业现代化、旅游产业化“四化”同步发展，胜利完成了“十二五”规划确定的各项目标任务。

这五年，是安顺聚力提速发展、综合实力更加增强的五年。

预计，地区生产总值由2010年的232.92亿元增加到2015年的620.36亿元，年均增长14.9%，是2010年的2.67倍。固定资产投资由111.34亿元增加到1300亿元，年均增长63.5%，是2010年的11.68倍。规模以上工业增加值由54.85亿元增加到150亿元，年均增长14.5%。财政总收入和一般公共预算收入分别由34.5亿元和19.84亿元增加到97.6亿元和69.86亿元，年均增长23.1%和28.6%。社会消费品零售总额由77.65亿元增加到155.82亿元，年均增长14.9%。金融机构人民币存、贷款余额分别由362.19亿元和229.65亿元增加到852.75亿元和610.27亿元，年均增长18.7%和21.6%。城镇和农村居民人均可支配收入分别由14504元和3526元增加到22957元和7436元，年均增长9.6%和16.1%。在全省经济发展综合测评中，我市排位从挂末上升到第五位。

这五年，是安顺加大投资力度、发展基础更加夯实的五年。

项目投资增长迅速。累计完成固定资产投资3547.5亿元，是“十一五”的8.61倍。实施市级重大工程和重点项目1889个，完成投资1960亿元，其中，亿元以上项目910个，完成投资1710亿元。实施“5个100工程”项目1309个，完成投资1501.14亿元。

交通网络升级完善。完成交通建设投资484亿元。新增高速公路通车里程150公里，安普、惠兴、六镇高速建成通车，安紫、花安、普织高速快速推进，2013年率先在全省实现县县通高速。国省道和农村公路提等改造成效明显，建成通村油路（水泥路）4600公里，实现村村通油路（水泥路）。沪昆客专安顺段及境内3个站点加快建设，安六城际铁路开工建设。黄果树机场开通北京、上海、重庆、广州、昆明、杭州、丽江等地航线。

水利工程建设加快。完成水利建设投资78亿元。黄家湾水利枢纽工程获国家批准立项，马马崖一级水电站建成发电，黔中水利枢纽一期工程基本建成，石朱桥水库下闸蓄水，新场河水库等骨干水源工程顺利推进，纳井田、老营、猫洞河、红坪、三岔河、木拱河等水库开工建设。治理病险水库93座，新建堤防201公里，新增有效灌溉面积24.6万亩。累计解决55.86万农村人口饮水安全问题。

信息基础不断夯实。“三网融合”试点启动实施，与贵阳、遵义实现通信同城化，4G网络建设顺利推进，通信光缆线路总长达10.32万公里，行政村通宽带率达95.1%，自然村通电话率达100%，乡镇邮政网点运营全覆盖。西秀区列为国家首批信息消费试点城市和国家第三批智慧城市试点。

这五年，是安顺加快转型升级、产业结构更加优化的五年。

新型工业化加速推进。累计完成工业投资1039.52亿元，年均增长69.7%。新增规模以上工业企业247户。轻工业、民营工业增加值占工业增加值比重由8.6%、48.8%提高到24.8%、67.5%。民用航空产业基地、黎阳产业园区、西秀工业园区、普定循环经济工业基地列入省级新型工业化示范基地，镇宁轻工产业园被确定为“贵州省轻纺产业示范园”。园区总产值由91.87亿元增加到420亿元，园区规模以上工业企业由98户增加到307户。台泥水泥、华兴玻璃、久联民爆、天台山制药、顺立达纺织、汇景纸业等一批建材、化工、装备、医药、特色轻工项目建成投产。

农业现代化稳步提升。在确保粮食安全的基础上，蔬菜、茶叶、水果、烤烟、中药材、生态畜牧等特色优势产业不断壮大，粮经比由2010年的52:48调整为2015年的30:70，畜牧业增加值占第一产业比重连续位居全省第一，2015年达到30.5%。建成省级农业示范园区18个，乡镇农业园区实现全覆盖。全市农机总动力达195万千瓦，农机化综合水平提高到50%。农产品“三品一标”工作扎实推进，成功申报农产品商标879件、农畜地理标志产品8个。农业产业化经营水平大幅提高，培育了2个国家级、43个省级、193个市级重点龙头企业及1643个农民专业合作社。

现代服务业蓬勃发展。旅游基础设施和配套服务不断完善，旅游升级版成效明显。黄果树、龙宫等景区品质全面提升，新增格凸河、旧州、云峰八寨、多彩万象、兴伟石博园5个国家4A级旅游景区。成功举办系列国际赛事和节庆活动，实现旅游与文化、体育等融合发展。黄果树智慧旅游建设走在全省前列，线上交易额达1.12亿元。全市累计接待游客1.36亿人次，年均增长28.4%，实现旅游总收入1312亿元，年均增长29.8%。金融保险、交通物流、邮电通信、信息服务、酒店餐饮等服务业快速发展，服务业聚集区建设成效明显。2015年第三产业实现增加值320.17亿元，是2010年的3.08倍。

新兴产业方兴未艾。大数据信息产业初见成效，软通动力、蚂蚁众创空间、黔中创客小镇、黄果树电商产业园正式运营。大健康医药产业快速发展，百灵制药扩能升级，苗立克、三力制药、中隆盛达等大健康产业项目落地建设，成功组建安顺市大健康医药投资有限公司。新型建筑建材业加快发展，建筑企业数量和资质迅速提升，石材产业欣欣向荣，晨春、天韵、玉腾等石材企业发展壮大，西南国际石材交易博览中心开工建设。新能源产业稳步发展，永宁、普屯坝风电场建成发电，绿色动力垃圾发电项目建成投产。

这五年，是安顺加强规划建设、城乡变化更加显著的五年。

规划引领调控有序。大力推进多规融合，组织编制《安顺市城市总体规划修编（2015-2030）》，市县镇村四级规划实现全覆盖。中心城区近期建设区域控规覆盖率100%，城市重要区域和重要节点规划设计水平进一步提高。完成各县城总体规划编制，各县城近期建设区域控规覆盖率95%以上。规划执法力度不断加大，拆除违法建筑148.3万平方米。

城市面貌焕然一新。贵安大道、安普大道、二环路等城市骨干路网建成通车，中心城区道路总长从193.2公里增加到311.2公里，中心城区面积拓展到90平方公里。万绿城、多彩万象等城市综合体建成运营。市政配套设施不断完善，城市综合承载能力明显增强。平坝撤县设区，普定、镇宁与中心城区同城化发展步伐加快，关岭顶云新区、紫云新城建设全面推进。全市城镇化率达到44%，比2010年提高9个百分点。成功获批国家新型城镇化综合试点。

城乡环境更加优美。建立健全城市管理体制机制，推行数字化管理，大力实施绿化、美化、亮化、净化工程，建成一批停车场、人行天桥和地下通道。中心城区、县城饮用水源地水质达标率分别为100%、95.8%，城镇污水处理率91.3%，城乡垃圾无害化处理率85.2%。深入开展“五创”和“整脏治乱”工作，获评省级文明城市、卫生城市、园林城市、双拥模范城，获全国文明城市提名。

村镇建设欣欣向荣。“8+X”工程项目扎实推进，建成旧州、夏云、白岩、格凸河等一批特色示范小城镇。西秀区和夏云镇获全国第三批城镇发展改革试点，旧州镇获全国建制镇示范试点，“镇园合一”、“1+N”镇村联动建设模式在全省推广。深入开展“四在农家·美丽乡村”创建活动，大力实施基础设施建设六项行动计划，涌现出小河湾等一批特色新村，滑石哨、桃子、浪塘荣获“中国最美休闲乡村”称号。

这五年，是安顺强化保护治理、生态环境更加优美的五年。

生态建设成效明显。扎实推进“绿色安顺”建设三年行动计划，大力实施退耕还林等林业重点工程，完成营造林133.87万亩，森林覆盖率达到47%以上。建成18个城区山体公园、小型游园。邢江河国家级湿地公园启动试点建设，虹山湖获批省级湿地公园。治理小流域8条，关闭地下机井16口，治理水土流失面积667平方公里，综合治理石漠化面积507平方公里。

节能减排持续推进。开展“六个一律”环保执法行动，大力实施工矿企业脱硫脱硝改造，推进煤改气工程，淘汰燃煤锅炉91台，单位GDP能耗下降15个百分点。削减二氧化硫10.87万吨、氮氧化物1.88万吨、化学需氧量4100吨、氨氮520吨，全面完成节能减排各项目标任务。城市环境空气质量达标率99.6%。

这五年，是安顺深化改革开放、发展活力更加强劲的五年。

深化改革蹄疾步稳。国企改革有序推进，组建安投公司等一批平台公司。军民融合深度发展，云马、风雷民品生产逐步扩大。大力扶持民营企业创新发展，民营经济增加值占生产总值比重达64%。混合所有制改革走在全省前列，组建安顺油脂集团等一批混合所有制企业。财税、金融、教育、文化、医疗卫生等体制改革扎实推进。成功创建首个农村金融信用市。西秀、紫云农村信用社改制为农村商业银行。普定、镇宁获批国家县城基础设施投融资体制改革试点县。家喻型材、新金秋科技在新三板成功上市。不动产登记工作有序开展。农村综合改革稳步推进，普定县发放全省第一本《农村土地流转经营权证》，率先实施土地流转经营权抵押贷款工作。西秀区和黄果树景区、格凸河景区纳入全省旅游体制机制改革试点，黄果树、龙宫实现一体化营销。强化基层管理职能，组建“乡规委”和“法工委”。

对外开放不断拓展。招商引资工作扎实推进，在广州、成都等地新设立8个招商分局，积极参与酒博会、厦洽会、西博会等系列招商活动，先后赴新加坡、迪拜、台湾、香港等地举行招商引资推介，成功引进项目1230个，实际到位资金2606.84亿元。争取上级支持力度明显加大，累计获得国家各类专项资金、专项基金和财政转移支付533亿元，是“十一五”的2.77倍。与贵阳、贵安新区战略合作深入开展。与发达地区交流合作不断加强，青岛新一轮对口帮扶工作全面推进，安顺-青岛产业园区建设初见成效。率先在深圳前海设立金融窗口，开展境外融资业务。

创新能力日益增强。综合科技进步水平指数达49.7%，每万人人才资源数987人。新增省级工程技术研究中心8个、博士后工作站3个、院士工作站3个，认证国家高新技术企业22个、国家创新型企业1个。获评全国科技进步先进市、国家知识产权试点优秀城市，成功创建安顺国家农业科技园区。

这五年，是安顺聚焦民生改善、社会事业更加进步的五年。

扶贫攻坚步伐不断加快。全面启动“32768”扶贫攻坚行动计划，总结推广“猴场模式”、“秀水五股”等扶贫经验，大力实施精准扶贫。全市农村扶贫对象从2011年的74.65万人减少到2015年的34.42万人，贫困发生率从30.8%下降到13.9%，先后有2个县、40个贫困乡镇实现“减贫摘帽”。

社会保障体系逐步完善。全市城镇新增就业18.37万人，城镇登记失业率控制在4.2%以内。实现养老、医疗、生育、工伤、失业五项社会保险制度全覆盖，城镇职工、城乡居民基本养老保险参保率分别达到87%和80%，城乡三项基本医疗保险参保率达到95.5%，大病保险制度全面实施。城市和农村低保分别从220元/月、1219元/年提高到464元/月、2605元/年。农村敬老院实现乡镇全覆盖。新建保障性住房12.6万套，建成6.2万套，完成农村危房改造10.86万套。

各项社会事业全面发展。全面实施农村义务教育阶段学生营养餐改善计划，教育“9+3”计划圆满收官，“4+2”突破工程加快推进，全面完成5所中职学校“百校大战”项目建设，安顺学院改扩建扎实推进，安顺职院、市二中顺利搬迁，新建成五所县级高中。义务教育巩固率达86%，学前教育三年毛入园率、高中阶段毛入学率、高等教育毛入学率分别从57%、47%、18.2%提高到85.4%、85.9%、32.1%。公共文化服务水平不断提高，建成一批电子阅览室、农家书屋、文化信息资源工程点，实现县县有数字影院、乡乡通有线电视、村村通广播电视。“双阅读”模式引领全民阅读广泛开展。新增国家级文物保护单位5处、省级文物保护单位3处，云山屯等34个传统村落纳入全国传统村落保护发展名录。普定县被命名为“全国文化先进县”。启动黄果树·屯堡景观世界自然与文化双遗产申报工作。全民健身运动广泛开展，市奥体中心基本建成。覆盖市县乡村四级的医疗卫生服务体系进一步完善，市人民医院新院、市儿童医院建设完工，全市千人医疗床位数达4.27张，比2010年增加1.8张。人口计生工作扎实开展，全面完成“双降”目标任务。

平安安顺建设扎实推进。深入开展社会治安综合治理，严厉打击各类违法犯罪，群众安全感和满意度分别从82.9%、87.1%提高到95.9%、97.4%。基层基础不断夯实，建成300平方米以上村级活动场所320个。总结推广关岭“上关经验”、普定“五民工作法”等做法，强化社会矛盾纠纷排查疏导，畅通信访渠道，及时化解不稳定因素。安全生产责任体系不断完善，安全事故起数和死亡人数持续“双降”。消防、应急处置、防灾减灾、食品药品监管、重大疾病防控等公共安全工作力度不断加大。高度重视隐蔽战线工作。法治建设和精神文明建设深入推进。全面落实民族宗教政策。国防建设、双拥、老干、档案、史志、外事、侨务、对台、人防、气象等工作持续加强。

这五年，是安顺推进依法行政、服务效能更加提升的五年。

自觉接受市人大的法律监督、工作监督和市政协的民主监督，办理人大代表建议388件，政协提案958件，办复率100%。认真听取各民主党派、工商联和无党派人士的意见建议。大力推进政务公开，提高政府透明度。全面推进民生监督，强化审计监督，主动接受新闻舆论和社会公众监督。深入扎实开展党的群众路线教育实践活动和“三严三实”专题教育。制定公布市直部门行政权力清单、责任清单。严控“三公经费”，从2013年起压缩行政经费5%用于发展教育。市、县政务服务中心建成运行，行政部门公共资源交易项目全部集中在市公共平台交易，电子政务建设加快推进。严厉查处各类违纪违法行为，勤政廉政建设取得新成效。

各位代表，刚刚过去的2015年是全面完成“十二五”规划的收官之年，是全面深化改革的关键之年，也是全面推进依法治市的开局之年。一年来，我们勇于探索、大胆实践，全力打好“一二四”攻坚战，经济社会继续保持快速发展的良好势头。预计，全市地区生产总值增长13.6%，一般公共预算收入增长19.5%，规模以上工业增加值增长11.8%，固定资产投资增长30%，社会消费品零售总额增长11.8%，城镇和农村居民人均可支配收入分别增长9.1%和11.5%，“十件实事”全面完成。我们全力推进国家新型城镇化综合试点，成功举办全省第十届旅游产业发展大会、全省项目观摩会、首届贵州（安顺）国际石材博览会。安顺获评“2015中国最美丽城市”，黄果树荣获“全球低碳生态景区”称号。全市上下干事创业蔚然成风、各项工作创先争优、各个领域亮点纷呈。

五年来取得的历史性成就，是市委坚强领导和科学决策的结果，是市人大、市政协有效监督和大力支持的结果，是全市上下解放思想、同心同德，共谋发展、团结奋斗的结果。在此，我谨代表市人民政府，向全市各族人民，向各位代表、政协委员，向各民主党派、工商联、无党派人士、人民团体，向驻安人民解放军、武警消防官兵、公安政法干警、中央和省驻安单位，向老同志和所有关心支持安顺经济社会发展的各界人士和朋友们，表示崇高的敬意和衷心的感谢!

各位代表，五年的成绩令人振奋，积累的经验弥足珍贵，创造的精神财富影响深远。回顾“十二五”，我们深切体会到：必须始终坚持发展第一要务。以经济建设为中心不动摇，理清发展思路不折腾，保持较快增速，加快做大总量，着力提升发展质量和效益。必须始终坚持守住生态底线。既要金山银山，更要绿水青山，以生态文明理念引领经济社会发展，保护好天蓝、山青、水净、地洁的生产生活环境。必须始终坚持改革开放。靠改革增活力，靠开放促发展，靠创新解难题，促进内部活力与外源动力竞相迸发。必须始终坚持依法行政。依法调整政府与市场、企业、社会的关系，做到职权法授、程序法定、行为法限、责任法究。必须始终坚持以人为本。把改善民生作为工作的出发点和落脚点，努力使各项工作经得起实践、人民和历史的检验。必须始终坚持苦干实干。树立“弱鸟先飞”意识，发扬“滴水穿石”精神，倡导“马上就办”作风，跳起来摘桃子，一以贯之接着干，用广大干部的辛苦指数换取人民群众的幸福指数。

在充分肯定成绩的同时，我们也清醒地认识到存在的问题和不足，主要是：经济总量小、县域经济实力弱；传统产业转型升级慢，新兴产业支撑不足；工业对资源依赖重，产业链短、带动力弱；产业项目占比较低，缺乏引领发展的大项目、好项目；市场主体少，缺乏支撑发展的大企业；生产要素成本上升，企业融资难融资贵问题突出；生态建设欠账多，资源约束和环境压力大；农村贫困面大、贫困程度深、贫困人口多，脱贫攻坚任务艰巨；城市功能不够完善，城市特色不够鲜明；社会治理水平不高，公共产品和公共服务供给不足；少数公职人员不作为、慢作为、乱作为的现象依然存在，勤政廉政建设还需进一步加强。我们必须切实增强忧患意识、创新意识、宗旨意识、使命意识，采取积极有效措施，不断加以解决。

二、“十三五”时期

经济社会发展总体要求

市委三届八次全会明确指出，“十三五”时期，我们将坚持创新、协调、绿色、开放、共享五大发展理念，围绕“四个全面”战略布局，把握“守底线、走新路、奔小康”要求，坚持“主基调、主战略”，大力实施大扶贫、大数据战略行动，高举国家新型城镇化综合试点旗帜，持续打好“一二四”攻坚战，推进“四个轮子”一起转，加强“五型城市”建设，成为全国新型城镇化示范区、黔中经济区重要增长极、生态文化旅游融合发展示范区和大健康医药产业创新示范区，提速发展走新路，建设秀美新安顺，全面建成小康社会。

“十三五”时期，我市经济社会发展的预期目标是：地区生产总值年均增长14%左右；规模以上工业增加值年均增长12%以上；固定资产投资年均增长25%以上；社会消费品零售总额年均增长13%以上；一般公共预算收入年均增长15%左右；接待游客人次年均增长25%以上，旅游收入年均增长27%以上；城镇居民人均可支配收入年均增长13%左右，农村居民人均可支配收入年均增长15%以上；城镇新增就业累计完成29万人以上；城镇化率达到61%；森林覆盖率达到60%；单位生产总值能耗下降目标和主要污染物减排指标达到国家和省的要求。

未来五年，我们将坚持五大发展理念，努力建设总量大、产业强、城乡美、百姓富、社会稳的秀美新安顺。

（一）坚持创新发展，全力打造黔中经济区重要增长极

创新是引领发展的第一动力，必须聚焦创新驱动，壮大市场主体，完善产业体系，努力实现弯道取直、后发赶超。

坚决守住速度底线。坚持发展第一要务，千方百计稳增长强动力，保持快速发展的良好势头。加快推进结构性改革，充分发挥市场在资源配置中的决定性作用。坚定不移抓好固定资产投资和招商引资，优化投资结构，促进人才、技术、知识、信息等要素升级。培育新型消费热点，促进消费结构升级。发展外向型经济，扩大进出口贸易规模。千方百计保持两位数的经济增长速度，不断壮大经济总量。

构建特色优势产业体系。大力实施工业强市战略，做大做强工业经济，充分发挥和挖掘一产、三产的优势和潜力，着力构建多元发展、多极支撑的现代产业体系。实施大数据战略行动，加强信息基础设施建设，推进大数据广泛深度应用，实施“互联网+”行动计划，拓展网络经济发展新空间。大力发展以大健康为目标的医药养生产业、以民族和山地为特色的文化旅游业、以节能环保为主导的新型建筑建材产业。推进煤炭、电力、化工、轻工等传统产业转型升级，大力发展飞机、汽车等装备制造业。加快转变农业发展方式，加强农业基础设施建设，调整优化农业产业结构，积极发展农产品精深加工，完善农产品市场流通、质量安全和农业科技服务体系，推进农业“接二连三”融合发展。大力提升现代服务业发展水平，加快发展现代金融、现代物流、电子商务等生产性服务业和健康服务、家庭服务、养老服务等生活性服务业，推动房地产业健康有序发展。

培育壮大市场主体。深入实施“5个100工程”，加大产业园区特色主导产业和企业集群培育，提高园区经营管理水平，促进园区提质升级，建成千亿级园区3个、百亿级园区2个。重点实施“百企引进”、“千企改造”工程和“双培育”、“双退出”行动计划。深入实施工业“百千万”工程，扎实开展服务企业、服务项目“双服务”行动。大力扶持龙头企业、骨干企业、上市企业，培育引进一批平台型企业和带动能力强的大企业，聚集发展一批中小企业，支持小微企业、个体工商户发展壮大，形成大企业“顶天立地”、中小企业“铺天盖地”、新兴企业“勇闯天地”的生动局面。

大力实施创新驱动。加强科技创新平台建设，建成一批重点实验室、工程技术研究中心、企业技术中心、众创空间等创新平台。建立以企业为主体、市场为导向、产学研相结合的科技创新体系，支持企业开展关键技术研发，提高企业核心竞争力。强化人才支撑，健全完善人才服务保障体系，着力培养引进复合型、创新型人才和企业家队伍。加强自主知识产权创造和保护。深化科技体制改革，推动从研发管理向创新服务转变，优化创新环境，推动大众创业、万众创新，最大限度地发挥创新对经济社会发展的支撑引领作用。

（二）坚持协调发展，全力打造全国新型城镇化示范区

协调发展是持续健康发展的内在要求，推进新型城镇化进程，必须坚持区域协调发展，提高城镇建设管理水平，促进发展平衡，增强发展后劲。

加快新型城镇化进程。建立农业转移人口市民化成本分担机制。不断完善和落实户籍制度改革“1+N”文件体系，统筹推进土地、财政、教育、就业、医疗、养老、住房保障等配套改革，促进基本公共服务均等化。放宽市政公用事业市场准入，建立多元化可持续的城镇化投融资机制。深入推进行政区划调整，加快普定撤县设区，积极推进镇改办、乡改镇、村改居工作。结合大扶贫战略行动，推广“一分三向”安顺新型城镇化模式，确保完成试点任务，努力建成“五型城市”。

推进城镇建设管理。统筹推进城镇路网、供水、供电、供气、排水、垃圾污水处理以及交通枢纽站场、停车场、加气站、充电桩、防灾避险等基础设施建设，开展“海绵城市”和地下综合管廊建设，增强城镇综合承载能力。大力提升城市文化品位，彰显城市文化特色，规划建设一批特色文化景观。加强城镇管理，提高精细化管理水平。成功创建全国文明城市和国家卫生城市。

促进区域协调发展。全面落实主体功能区战略，优化空间开发格局，构建中心城市、县城和小城镇合理分工、功能互补、协同发展的城镇体系。实施城镇化“1151”工程，依托主城区、平坝、普定的建设发展，做大做优区域性中心城市，建成百万人口生态旅游城市，形成安顺-贵安-贵阳城市组群的重要增长极。不断完善县城和重点镇产业体系，做大做强县域经济，建成50个省市级示范小城镇。深入开展“四在农家·美丽乡村”建设，建成100个省级特色美丽乡村。

加强基础设施建设。完善现代综合交通运输网络。推动贵阳至兴义、黄桶至百色铁路和贵阳-贵安-安顺城际铁路建设。推进紫云至望谟、都匀至香格里拉高速公路安顺段建设。加快安顺与贵安新区互联互通道路建设，加大国省县乡道改造力度，提高客运通达率。实施黄果树机场改扩建工程。建设现代化内河水运和旅游航运体系，打通黔中出海通道。完善现代水利工程网络，实现每个县城有一座以上中型水库或几座重点小型水库，每个乡镇有稳定的供水水源。加快完善信息基础设施，全市行政村通宽带率达到100%。

（三）坚持绿色发展，全力打造生态文化旅游融合发展示范区

树立绿水青山就是金山银山的理念，正确处理开发与保护的关系，不断推进生态、文化、旅游融合发展，让现代文明与生态文明交相辉映。

突出生态建设。大力实施“绿色安顺”建设三年行动计划，推进封山育林、植树造林和退耕还林，建立保护森林资源的长效机制。加快湿地公园建设，保护生物多样性。强化水土保持，实施坡耕地治理、小流域综合治理、石漠化综合治理等工程。开展城乡绿化美化，建设高铁、高速、城际干道沿线绿色景观长廊，实施山体公园建设，积极推进农村环境综合整治，改善城乡环境质量，成功创建国家园林城市。

加强环境保护。加大对水、大气、土壤污染的防治工作，执行环境影响评价制度，严格项目准入许可。完善以预防为主的环境管理制度，建立环境保护投入市场化机制，探索推行资源有偿使用制度。大力发展循环经济，推广清洁能源，推进企业清洁化生产、园区生态化改造，建成一批新物质能源项目和生态工业园区。加快淘汰落后产能、过剩产能，扎实推进主要污染物总量减排工作。倡导绿色发展理念，使生态文明成为全社会的共同追求和自觉行动，成功创建国家环保模范城市。

加快旅游发展。大力实施山地旅游发展战略，推进旅游与文化、体育、农业、工业等相结合，形成新业态新产品，提升旅游附加值。充分挖掘屯堡、民族、红色、三线等历史文化内涵，大力发展文化产业，促进与旅游业深度融合发展。推进黄果树·屯堡景观申报世界自然与文化双遗产和关岭化石群申报世界自然遗产工作。加快全域旅游基础设施建设，创新设计旅游产品，增强游客参与度和体验度，构建快旅慢游体系。支持旅行社等旅游企业加快发展，大力拓展旅游市场，推进以黄果树为重点的智慧旅游建设。充分发挥生态优美、气候凉爽、景点富集等优势，大力发展休闲度假、体育健身、科考教育、避暑养生、民俗文化和乡村旅游，努力实现全域景区化、景区精品化、文旅一体化、业态多样化、营销数字化，成功创建国家级全域旅游示范区。

（四）坚持开放发展，全力打造内陆开放型经济新高地

坚持以开放倒逼改革，以改革促进开放，始终把改革贯穿经济社会发展各领域，积极拓展开放新空间，推动开放型经济迈向更高层次。

深化改革激发活力。深化国企改革，加快完善现代企业制度。建立健全平台公司运作机制，有效防范风险。大力发展混合所有制经济，鼓励民营资本进入公共领域，到2020年民营经济比重提高到70%。深化行政管理体制改革，推进简政放权向纵深发展。深化商事制度改革。推进军民深度融合发展改革，引导国有资本和民营资本参与民用航空产业基地发展。深化投融资体制改革，推广PPP模式，引导更多社会资本参与产业发展和基础设施建设。深入推进农村产权制度改革。深化社会事业体制改革，健全和完善教育、医疗、养老、就业、住房等社会保障制度。

搭建平台扩大开放。加快推进以国家新型城镇化综合试点为引领，以民用航空产业基地、黎阳高新区、全域旅游示范区、文化产业示范园区和农村金融信用市为重点的“1+5”平台建设，打造开放新高地，培育经济增长极。加快保税仓、保税物流园区建设，推进黄果树机场航空口岸、黄桶-幺铺物流园陆路口岸建设，力争启动综合保税区、海关、检验检疫分支机构建设，规划建设临空经济区。

加强合作强化招商。深化与贵阳市、贵安新区的战略合作。认真谋划“走廊产业经济带”，积极参与沪昆高铁、沪昆高速经济带建设，构建区域合作发展新格局。主动融入“一带一路”、长江经济带、珠江-西江经济带发展。加强与青岛等发达城市和先进地区的交流合作。借力生态文明贵阳国际论坛、酒博会、数博会、民博会、石博会、旅发会、世界山地旅游大会等重大活动平台，围绕大数据、大健康、电子商务、新型建筑建材等领域开展招商引资。优化发展环境，积极引进战略投资者，吸引更多外部先进要素集聚安顺。

（五）坚持共享发展，全力推进全面建成小康社会

着力解决民生难题，大力实施大扶贫战略行动，推进基本公共服务均等化，让人民群众有更多全面小康的获得感、幸福感。

实施大扶贫战略行动。扶贫开发是重大政治任务，脱贫攻坚是第一民生工程。坚持科学治贫、精准扶贫、有效脱贫，扎实推进“32768”扶贫攻坚行动计划，落实好“1+10”配套政策措施。建立完善市县乡村四级联动扶贫工作机制。发挥群众主体作用，提高自我发展能力。实施发展生产、易地搬迁、生态补偿、发展教育、社保兜底“五个一批”工程，确保每年实现6万人以上贫困人口脱贫，到2019年现有贫困人口全部实现脱贫，决不让一个地区、一个民族、一个困难群众在同步小康中掉队。

多渠道增加群众收入。健全促进就业和鼓励创业机制，加强职业技能培训，多渠道开发就业岗位，促进高校毕业生、退役军人、困难群体就业，鼓励群众通过勤劳致富改善生活。认真落实收入分配改革的政策措施，完善最低工资和工资支付保障制度。鼓励和引导农民以土地承包经营权、宅基地使用权、林权等开展股份合作，促进农民持续增收。继续抓好“保供稳价”和“菜篮子工程”，确保居民消费价格总水平保持基本稳定。

提高公共服务水平。稳步提高“五险一金”社会保障水平。整合资源加快城乡一体的社会保障制度建设，完善城乡养老保险、医疗保险制度的衔接和转移接续政策。坚持教育优先发展，全面提高教育质量，着力促进教育公平。大力实施教育提升工程，实现县域义务教育基本均衡和基本普及十五年教育。加快发展现代职业教育，提高高校办学规模和水平，积极发展民办教育。建设“健康安顺”，深化基层医疗卫生体制改革和公立医院改革，推进医疗卫生计生标准化、信息化和人才队伍建设，构建“15分钟城市社区健康服务圈”和“30分钟乡村健康服务圈”。坚持计划生育基本国策，全面落实放开二孩生育政策，促进人口均衡发展。积极应对人口老龄化。加快基层社区和农村公共服务能力建设。

不断创新社会治理。加快建设法治政府，切实提高政府公信力。充分发挥政府主导作用，强化系统治理、依法治理、综合治理和源头治理，进一步提升社会治理能力。加强精神文明建设，践行社会主义核心价值观。推进社会信用体系建设。加强社会稳定风险评估，强化信访工作，完善矛盾纠纷排查调处机制，有效预防和化解各类社会矛盾。实施最严格的食品药品监管，严厉打击制假售假行为。推进国防教育、国防动员、国防后备力量和人民防空建设，启动新一轮全国双拥模范城创建工作。强化安全生产责任体系、隐患排查治理体系和应急救援保障体系建设。树立“大平安”理念，着力打造“平安安顺”升级版，进一步提升人民群众安全感和满意度。

各位代表，提速发展走新路在全市上下已形成共识，建设秀美新安顺已成为人民群众的强烈愿望，“十三五”的蓝图已经绘就，序幕已经拉开，号角已经吹响，我们对未来的发展充满信心。我们坚信，在市委的坚强领导下，在全市各族人民的共同努力下，今后五年的宏伟目标一定能够实现，一个更加繁荣、更加和谐、更加幸福、更加秀美的新安顺必将展现在黔中大地!

三、2016年重点任务

今年是全面建成小康社会决胜阶段的开局之年，做好今年各项工作，实现“十三五”开门红，意义十分重大。2016年全市经济社会发展的主要目标是：地区生产总值增长14%左右；规模以上工业增加值增长12%；固定资产投资增长25%；一般公共预算收入增长13%；社会消费品零售总额增长13%；城镇和农村居民人均可支配收入分别增长13%和15%；城镇登记失业率控制在4.2%以内；居民消费价格涨幅3%左右；城镇化率达到47.5%；完成省下达的节能减排目标任务。

围绕实现上述目标，重点抓好以下十个方面工作：

（一）紧盯补短板抢先机，在大扶贫大数据战略行动上求突破

大力推进大扶贫战略行动。实施精准扶贫，按照“七个到村到户”和“六个精准”的要求，切实提高扶贫开发的针对性和实效性。加大投入力度，市级财政统筹安排2.5亿元用于脱贫攻坚，用好扶贫开发投资平台，整合各类扶贫资金、涉农资金投向扶贫脱贫，充分发挥金融机构在扶贫中的资金支持作用。认真落实“五主”、“五包”责任制，实施民营企业“千企帮千村”行动，广泛动员全社会力量，对贫困县、乡、村和贫困户实现帮扶全覆盖。建好用好“扶贫云”和“扶贫专线”。实施脱贫攻坚“五个一批”、“十一项行动”重大项目，实现产业扶持3.75万人、新增就业扶持5300人、教育扶持2.3万人、“两无”人员民政低保兜底16.56万人。镇宁自治县及13个贫困乡镇按省定标准实现摘帽，110个贫困村出列。

大力推进大数据战略行动。加快大数据基础设施建设，深入实施信息基础设施三年会战，加快4G组网、“宽带乡村”试点工作，完成信息基础设施投资10亿元。实施“互联网+”行动计划，培育5家以上“互联网+”示范企业。依托“云上贵州”，积极构建跨区域、跨部门、跨行业的数据汇聚交换平台，建设“旅游云”、“中小企业云”等专业云平台，在大数据应用水平上取得突破。积极引进扶持培育一批大数据优强企业，加快软通动力、国电南自、迷境生态等企业发展。大力推进智慧城市建设，提高城镇管理信息化水平。深入推进西秀区信息消费试点城市和智慧城市试点工作。推动大数据与大健康有机融合，实施大健康医药产业发展六项计划，推进“安顺健康云”和“智慧医疗”平台建设，支持百灵制药、兴东大健康产业园等项目建设发展。

（二）紧盯提速度增总量，在固定资产投资招商引资上求突破

强化项目建设。紧紧围绕“5个100工程”，谋划储备一批打基础、管长远的重大工程和重点项目。市级筹措经费5000万元以上，扎实推进项目前期工作，确保项目库投资规模保持在1.38万亿元以上。实施市级重大工程和重点项目466个，年度投资742亿元。以项目观摩会为抓手，全力做好项目培育调度和督促检查。完善土地保障机制，新增建设用地500公顷，优先保障重点区域和重点项目用地需求。拓宽投融资领域和途径，优化投资环境，吸引更多资金投向高新技术产业、新兴产业和现代服务业。产业投资占固定资产投资比重40%以上。

加大招商引资。强化招商引资项目库建设，储备亿元项目300个以上。发挥驻外招商分局的作用，不间断进行定点招商。加强与500强企业对接联络，围绕大数据、大健康等产业招大商、选好商。强化招商引资目标责任管理，切实抓好全程跟踪服务，确保招商引资项目到位资金增长25%，项目开工率80%以上。

（三）紧盯抓试点出成效，在加快新型城镇化建设上求突破

抓好新型城镇化综合试点。实施新型城镇化基础设施建设规划，明确“城镇村”的角色分工和发展方向，奠定人口转移基础。深化户籍制度改革，引导人口有序转移。加快建立农业转移人口市民化成本分担机制。实施新型城镇化融资规划，在更多领域推广PPP模式，全力做好县城基础设施投融资试点和建制镇示范试点工作。落实新型城镇化综合试点实施方案和新型城镇化发展规划，深入推进多规融合，加快产业发展和项目建设，着力解决好转移人口就业问题，初步形成“一分三向”新型城镇化安顺模式。

加强城镇建设管理。实施水道、慢道、轨道“三道”工程建设。加快棚户区改造，启动安顺历史文化街区改造项目，加快北部新区、高铁片区等城市新区建设，推进平坝高铁新区、普定新城、镇宁环翠新区、关岭顶云新区、紫云新城等一批重点新区建设。整合行政执法主体，推进城管综合执法。大力加强公共停车场建设，千方百计破解停车难问题，发展公共交通，建成智能交通管控平台，有效改善城区拥堵现象。加强医疗废弃物、建筑垃圾、餐厨垃圾处置，强化噪音污染治理。加大创建工作力度，提升市民文明素质，改善群众生活环境。

推进美丽乡村建设。大力推广“1+N”镇村联动建设模式，完成“8+X”项目投资13亿元以上。继续实施“四在农家·美丽乡村”六个小康行动计划项目。加强民族村寨、传统村落保护和发展。建成20个设施完善、产业兴旺、生态良好、环境优美、魅力独特、宜居宜业宜游的省级特色美丽乡村，每个乡镇至少打造一个市级水平的美丽乡村。

加快基础设施建设。完成沪昆客专安顺段及境内3个站点建设，加快推进安六城际铁路项目，积极配合做好贵兴铁路开工建设工作。建成花安高速，加快安紫、普织高速建设，开工建设紫望高速。加快建设宁谷经龙宫至黄果树旅游公路、开发区至镇宁城市快速干道。完成木紫、镇坝等国省道改造工程，推进黄桶至幺铺、关岭至花江、镇宁至黄果树、平坝东外环线、北斗湖路（安顺段）等骨干道路建设。完成中心城区客运东站、西站、北站建设，建成25个乡镇客运站，建制村通客率达到95%。加快光照、董箐等库区航运工程建设。大力实施水利建设“三年行动计划”，开工建设大洞口、偏坡、坝陵河、木桥等水库，加快推进黄家湾水利枢纽工程，力争建成新场河、纳井田、老营、猫洞河等水库。

（四）紧盯提质量增效益，在加快新型工业化发展上求突破

推进工业转型升级。实施重点工业项目100个，实现规模以上工业增加值170亿元。改造提升煤炭、电力、化工、特色轻工等传统产业，加快发展民用无人机等高端装备制造业。大力发展石材产业，着力推进西南国际石材交易博览中心建设，办好第二届石博会。推进兴贵保温砌块等新型建筑建材产业加快发展。支持垃圾发电、新能源汽车制造等节能环保项目健康快速成长。

提高园区发展水平。深化园区管理体制改革，推行“管委会+经营公司”模式。加强园区内容建设和功能定位，推动园区突出特色、错位发展，增强园区综合竞争力。完成园区工业投资300亿元，实现工业总产值480亿元。加快民用航空产业基地建设，推动西秀、普定、镇宁、关岭、紫云等园区发展，力争国家级黎阳高新区获批。

增强企业竞争力。积极培育发展“五上”企业，新增规模以上工业企业60家，培育壮大资质等级建筑企业，支持房地产开发企业健康发展，扶持限额以上批零住餐企业和服务业企业加快发展。继续实施“3个15万元”等政策，扶持发展微型企业1600户。支持贵飞、安大等军工企业加快发展，盘活已搬迁军工企业资产。积极为企业提供技术支撑、政策支持，帮助企业拓宽融资渠道，拓展市场。

（五）紧盯调结构创特色，在发展现代山地高效农业上求突破

多举措调整农业结构。深化“乡乡镇镇建农业园区”工程，种植粮食217万亩，种植蔬菜140万亩，新增种植茶叶15万亩、水果12万亩、中药材8万亩，收购烤烟22.4万担。加快山地生态畜牧业发展，实现肉蛋产量19万吨、水产品产量1.9万吨，畜牧业增加值占第一产业比重达到32%。扶持壮大农产品加工龙头企业，大力发展金刺梨、火龙果和生猪、肉牛、禽蛋等农畜产品精深加工。抓好特色农产品质量认证和商标注册，扩大“三品一标”生产规模。

多渠道增加农民收入。推进“科技入户”工程，强化农民技能培训。推动高校、职业院校与农业园区、龙头企业、产业基地联动，不断提升农村合作组织带头人、农民经纪人、种养大户的经营能力。发展壮大农村集体经济，培育一批家庭农场、农民专业合作社等新型农业经营主体。积极发展农村电子商务，促进“安货出山”，推进西秀、平坝电子商务进农村省级示范县建设。全面启动农村土地承包经营权、宅基地使用权和林权确权登记颁证工作，有序推进农村土地流转。充分依托山地生态优势，大力发展生态农业、休闲农业、观光农业、光伏农业，拓展农民增收渠道。

（六）紧盯兴业态促升级，在以旅游为龙头的服务业上求突破

加快旅游业态创新发展。推进黄果树、龙宫、屯堡旅游景区管理一体化进程，提升景区发展水平。整合全市旅游资源，打造安顺大黄果树山地全域旅游系列产品。启动黄龙湖旅游养生基地、马厂飞行小镇、格凸攀岩小镇等项目建设。大力发展乡村旅游，开展“屯堡文化汇”活动。继续办好黄果树瀑布节、格凸河攀岩、坝陵河低空跳伞、镇宁半程马拉松等节庆和赛事活动。全年接待游客4875万人次以上，增长25%，旅游总收入480亿元以上，增长27%。

推进旅游配套设施建设。依托高铁站点和重要交通节点，加快建设游客服务体系。完善道路旅游标识标牌，建成一批景区停车场、厕所等配套设施。提高景区通达性，开通中心城区到景区和景区之间的旅游直通车。新增黄果树机场对外航线3条以上。

大力发展现代服务业。深入实施服务业“2125”行动计划。加快推进黄桶-幺铺物流园区建设，支持川渝、豪德、黔中、梓涵等电商物流园区发展。合理布局商业网点，有序搬迁城区老旧市场，推动一批新型专业批发市场转型升级。规范管理二手车交易。加快现代金融、商务服务、研发设计、会展广告、信息技术服务等生产性服务业创新发展，推进商贸、家政、住宿餐饮、社区照料、医患陪护等生活性服务业加快发展。实现第三产业增加值增长16%。

（七）紧盯守底线优环境，在推进生态文明建设上求突破

加快生态建设步伐。大力实施封山育林、人工造林、退耕还林工程，植树造林41万亩，开展城乡公共绿地、交通沿线绿化建设，森林覆盖率提高到49%以上。启动安顺植物园建设，推进邢江河、虹山湖、黄果树白水河等湿地公园建设。治理水土流失面积150平方公里，综合治理石漠化面积102平方公里。

加大节能减排力度。积极化解过剩产能，淘汰落后产能。加强脱硫脱硝等环保设施运行管理。完成黄标车、老旧车和高污染燃料锅炉年度淘汰任务。加快调整能源结构，积极推行清洁生产，大力培育环保、新能源产业。完成全国再生资源回收利用试点项目。支持西秀区全省绿色能源示范县建设。

加强环境保护治理。强化环境执法监管，着力控制和减少主要污染物排放，确保环境质量和污染排放总量“双控”。大力提升环境监测能力，深入实施环保网格化监管，实现环境监管全覆盖。全面推进“河长制”，加大集中式饮用水源地保护力度，加快推进夜郎湖一级保护区居民搬迁，启动桂家湖备用水源地工程建设。深入推进城乡环境综合治理，着力提升城镇污水处理率和城乡生活垃圾无害化处理率。抓好秸秆禁烧综合利用。

（八）紧盯挖潜力搭平台，在深化改革扩大开放上求突破

加快推进重点领域和关键环节改革。深化国企改革，加强国有资产资源整合，提高经营管理水平，推动国有控股公司上市。实施提高民营经济比重“五年行动计划”，民营经济增加值占生产总值比重65%以上。在教育、卫生、文化、体育、旅游等领域积极探索混合所有制实现形式。推进资源要素价格改革，促进资源产权和产品自由流动。深入推进不动产统一登记制度改革。加快农信社改制工作，进一步完善农村金融信用体系。实现村镇银行县域全覆盖。深化城乡水务一体化改革和水利工程管理体制改革，开展农田水利设施产权制度改革。

进一步提高对外开放水平。加快“1+5”平台建设，力争取得重要突破。推进安顺经济技术开发区申报国家级开发区。推动安顺-青岛产业园建设取得明显成效。积极加强与东盟、中东、加拿大、瑞士、韩国、港澳台等国家和地区交流合作，举办针对性强的经贸交流活动，推动合作项目取得实质性进展。深入实施外贸进出口翻番计划，确保进出口总额增长20%，实际利用外资增长25%。

（九）紧盯惠民生促和谐，在推动社会事业发展上求突破

全力推进社会事业发展。大力实施“新两基”攻坚，确保实现全市基本普及十五年教育目标。加快推进义务教育薄弱学校改造工程，启动市一中改扩建工程，推进安顺学院改扩建二期工程建设，完成安顺职院整体搬迁。增强医疗卫生服务能力，全面推进县级公立医院改革，启动城市公立医院改革。推进“百院大战”等六大医疗卫生机构标准化建设工程，市人民医院新院、市儿童医院全面建成投用，支持贵航302医院创建三甲医院，加快推进市中医院建设，支持专科医院发展。建成市规划展览馆，加快推进市博物馆建设，启动市文化大剧院等场馆建设。实施文化惠民工程，大力繁荣文艺创作，推出一批优秀作品。提升全民科学素质。大力发展体育事业，承办好全省第三届农民运动会。推进就业创业促进工程，实施“雁归兴安”计划、“锦绣计划”。深入实施全民参保计划。统筹推进城乡社会救助体系建设。加快发展慈善、老龄、残疾人事业，进一步做好民族宗教、新闻出版、广播影视、防灾减灾、史志、文物、档案、外事、侨务、对台等工作。

全力提高社会治理能力。加强城乡基层服务网络建设，完善社区配套设施和服务功能，推行社区网格化管理，创建和谐社区。加强社区矫正工作，积极预防青少年违法犯罪。加强公共法律服务体系建设，进一步扩大法律援助覆盖面。更加关心和关爱留守儿童、空巢老人。完善安全生产责任体系，加强安全生产监管，促进安全生产形势持续平稳。加强应急管理，妥善应对突发公共事件。强化基层信访平台建设，推进网上受理信访制度，做好信访积案化解工作，推进信访法制化进程。加强社会治安防控体系建设，加快推进“天网”三期工程，深入实施“平安细胞工程”，进一步提高人民群众的安全感和满意度。

（十）紧盯转作风提效能，在加强政府自身建设上求突破

全面推进依法行政。严格按照法定权限和法定程序行使权力、履行职责。依法科学民主决策，健全重大行政决策规则，强化重大决策终身责任追究制度及责任倒查机制。严格规范公正文明执法，强化行政权力制约和监督。自觉接受市人大的法律和工作监督、市政协的民主监督、新闻舆论和社会公众监督。充分听取民主党派、工商联和无党派人士的意见建议，支持工青妇等群团组织依法按章开展工作。

全力提高服务效能。进一步简政放权，落实权力清单、责任清单，最大限度取消行政审批事项。全面推进政府信息公开。完善政务服务中心、公共资源交易中心、网上办事大厅功能，推进并联审批、网上审批和电子监察，压缩行政审批事项办理时限。推进电子政务，降低政务成本，提高办事效率。引入竞争机制，推广政府向社会力量购买服务。

切实转变工作作风。认真贯彻落实中央“八项规定”精神，坚决反对“四风”，忠实践行“三严三实”。加强公职人员思想素质建设，大力整治懒政怠政和为官不为现象，始终保持勇于担当、甘于奉献、肯于吃苦的良好精神状态，始终把为人民谋发展增福祉作为最大责任，始终把脱贫攻坚、同步小康使命扛在肩上，始终把群众冷暖忧乐放在心头。

着力加强廉政建设。增强各级干部廉洁自律意识，不断完善惩治和预防腐败体系，推动反腐倡廉制度创新。发挥审计监督作用，抓好政府性资金、投资项目和自然资源资产、稳增长政策措施的审计监督工作。加强行政监察，强化权力运行制约和监督，健全完善市级重点巡查制度，深入开展专项监察和民生监督工作，坚决查处不正之风和腐败问题，努力实现干部清正、政府清廉、政治清明。

各位代表，民生是为政之本。围绕保障改善民生，我们将继续办好十件实事：①减少农村扶贫对象6万人；完成精准扶贫建档立卡贫困户庭院硬化10万户；完成易地扶贫搬迁2.2万人。②城镇新增就业5.2万人，新增创业2400人，创业带动就业9600人。③城镇和农村居民最低生活保障标准在2015年基础上分别提高10%和18.6%。④新建改扩建标准化乡镇卫生院42所、标准化村卫生室200个；规范建设公办性质的标准化社区卫生服务中心10个；建成计生家庭服务中心5个。⑤建成农村义务教育学校标准化食堂100个。⑥建成村级污水处理设施20个。⑦完成虹山湖景观整治二期工程建设。⑧建成中心城区山体公园（游园）10座。⑨调整优化城乡客运线路，开通主城区至旧州旅游专线；全面完成贵安大道（主城区至平坝）、二环路公交站台建设。⑩新增主城区公共停车位1000个。

各位代表，没有比人更高的山，没有比脚更长的路，我们已经走进了“十三五”，踏上了新征程。时代赋予我们重任，人民寄予我们厚望。让我们在中共安顺市委的坚强领导下，紧紧依靠全市各族人民，抢抓机遇、开拓创新，提速发展、后发赶超，为实现全面建成小康社会宏伟目标而努力奋斗！
